# Liquiritin Attenuates Lipopolysaccharides-Induced Cardiomyocyte Injury via an AMP-Activated Protein Kinase-Dependent Signaling Pathway

**DOI:** 10.3389/fphar.2021.648688

**Published:** 2021-05-14

**Authors:** Shan-Qi Mou, Zi-Ying Zhou, Hong Feng, Nan Zhang, Zheng Lin, Xiahenazi Aiyasiding, Wen-Jing Li, Wen Ding, Hai-Han Liao, Zhou-Yan Bian, Qi-Zhu Tang

**Affiliations:** ^1^ Department of Cardiology, Renmin Hospital of Wuhan University, Wuhan, China; ^2^ Cardiovascular Research Institute of Wuhan University, Wuhan, China; ^3^ Hubei Key Laboratory of Metabolic and Chronic Diseases, Wuhan, China; ^4^ Department of Geriatrics, Renmin Hospital of Wuhan University, Wuhan, China

**Keywords:** liquiritin, AMPKα, septic cardiomyopathy, inflammation, apoptosis

## Abstract

**Background:** Liquiritin (LIQ) is a traditional Chinese medicine that has been reported to regulate inflammation, oxidative stress and cell apoptosis. However, the beneficial effects of LIQ in lipopolysaccharides (LPS)-induced septic cardiomyopathy (SCM) has not been reported. The primary goal of this study was to investigate the effects of LIQ in LPS-induced SCM model.

**Methods:** Mice were pre-treated with LIQ for 7 days before they were injected with LPS (10 mg/kg) for inducing SCM model. Echocardiographic analysis was used to evaluate cardiac function after 12 h of LPS injection. Thereafter, mice were sacrificed to collect hearts for molecular and histopathologic assays by RT-PCR, western-blots, immunohistochemical and terminal deoxynucleotidyl transferase nick-end labeling (TUNEL) staining analysis respectively. AMPKα2 knockout (AMPKα2^−/−^) mice were used to elucidate the mechanism of LIQ Neonatal rat cardiomyocytes (NRCMs) treated with or without LPS were used to further investigate the roles and mechanisms of LIQ *in vitro* experiments.

**Results:** LIQ administration attenuated LPS-induced mouse cardiac dysfunction and reduced mortality, based upon the restoration of EF, FS, LVEDs, heart rate, dp/dt max and dp/dt min deteriorated by LPS treatment. LIQ treatment also reduced mRNA expression of TNFα, IL-6 and IL-1β, inhibited inflammatory cell migration, suppressed cardiac oxidative stress and apoptosis, and improved metabolism. Mechanistically, LIQ enhanced the phosphorylation of AMP-activated protein kinase α2 (AMPKα2) and decreased the phosphorylation of mTORC1, IκBα and NFκB/p65. Importantly, the beneficial roles of LIQ were not observed in AMPKα2 knockout model, nor were they observed *in vitro* model after inhibiting AMPK activity with an AMPK inhibitor.

**Conclusion:** We have demonstrated that LIQ exerts its protective effects in an SCM model induced by LPS administration. LIQ reduced inflammation, oxidative stress, apoptosis and metabolic alterations via regulating AMPKα2 dependent signaling pathway. Thus, LIQ might be a potential treatment or adjuvant for SCM treatment.

## Introduction

Sepsis is a life-threatening inflammatory disease and a major cause of death among patients in intensive care units due to infection-induced multiple organ failure (Rossaint and Zarbock, 2015). Septic Cardiomyopathy (SCM) characterized by cardiac dysfunction (Rello et al., 2017) has high mortality and morbidity in sepsis patients. Even though mechanisms involved in SCM have not been fully elucidated, SCM appears to be the result of inflammatory bursts, apoptosis, dysregulated energy metabolism and oxidative stress (Beesley et al., 2018; Luo et al., 2020; Tan et al., 2021). Strategies to attenuate SCM based upon inflammation, oxidative stress and energy metabolism have not been successful clinically (Rello et al., 2017). Thus, there remains an urgent need to develop new strategies to treat sepsis-induced SCM.

Natural flavonoids have been considered as potential treatments for sepsis and SCM because of their multi-biological activities containing anti-oxidative stress, anti-inflammation and depressing cell apoptosis (Kwon et al., 2010; Birari et al., 2011; Wang et al., 2011; Gaur et al., 2014). Recently, *Glycyrrhiza uralensis Fisch* has been studied because it contains several flavonoids such as glycyrrhizic acid, iso-glycyrrhizin and Liquiritin (LIQ) (Cantelli-Forti et al., 1994; Montoro et al., 2011; Farag et al., 2012; Yu et al., 2015). Recent studies have shown that LIQ could inhibit LPS-induced inflammatory cytokine expression in LPS treated glial cells and tert-butyl hydrogen peroxide treated mouse liver (Yu et al., 2015), improve ischemia-reperfusion associated neuro-injuries through its antioxidant and anti-apoptotic activities (Sun et al., 2010), and attenuate ultraviolet light-induced cell damage (Li et al., 2018).

However, it remains to be determined whether LIQ could exert a protective role in LPS-induced SCM mouse model. Thus, this study evaluated the effect of LIQ on the pathogenesis of SCM and clarified the underlying mechanisms.

## Materials and Methods

### Ethics Statement

This study was approved by the Animal Care and Use Committee of Renmin Hospital of Wuhan University (approval number: WDRX-2018K010). All studies were carried out in accordance with the Guidelines for the Care and Use of Laboratory Animals published by the United States National Institutes of Health (NIH Publication, revised 2011).

### Main Reagents

LIQ was purchased from Shang Hai Winherb Medical Science CO., Ltd. (White crystalline powder, purity >98%). LPS was purchased from Sigma-Aldrich (St. Louis, MO, United States). Compound C (S7840) was purchased from Selleck (Shanghai, China). Primary antibodies including anti-mTOR (T-mTOR, 2983), anti-phospho-mTOR (p-mTOR, 2971), anti-GAPDH (2118), anti-Bax (2772), anti-Bcl-2 (2870), anti-phospho-JNK (p-JNK, 4468), anti-JNK (T-JNK, 9258), anti-phospho-ERK (p-ERK, 4370), anti-ERK (T-ERK, 4695), anti-phospho-p38 (p-p38, 4511), anti-p38 (T-p38, 9212), anti-phospho-AKT (p-AKT, 4691), anti-AKT (T-AKT, 4060), anti-phospho-GSK3β (p-GSK3β, 9323), anti-GSK3β (T-GSK3β, 9315), anti-phospho-STAT3 (p-STAT3, 9136), and anti-STAT3 (T-STAT3, 9139) were purchased from Cell Signaling Technology. Primary antibodies, including anti-phospho-AMPKα2 (p-AMPKα2, ab109402), anti-AMPKα2 (T-AMPKα2, ab3760), anti-phospho-IκBα (ab133462), anti-IκBα (ab7217), anti-phospho-p65 (ab194726), and anti-p65 (ab16502) were obtained from Abcam (Cambridge, United Kingdom). Secondary antibodies were obtained from LI-COR Biosciences. The cell counting kit 8 (CCK-8) was purchased from Dojindo Molecular Technologies (Rockville, MD, United States), and the Cu/Zn-SOD, Mn-SOD and NADP+/NADPH assay kits were purchased from Beyotime (Shanghai, China).

### Animals and Lipopolysaccharides-Induced Septic Cardiomyopathy Models

C57BL/6J mice (male, weighing 23.5–25.5 g, and aged 8–10 weeks) were purchased from the Institute of Laboratory Animal Science, Chinese Academy of Medical Sciences (CAMS) & Peking Union Medical College (PUMC) (Beijing, China), and were kept in specific-pathogen-free conditions in the Cardiovascular Research Institute of Wuhan University. Mice had access to food and water freely and were housed with a 12 hour light/dark cycle.

Mice were randomly divided into 6 groups, the CON group (saline), LIQ80 group (80 mg/kg/d LIQ), LPS group (10 mg/kg LPS), LPS + LIQ20 group (LPS+20 mg/kg/d LIQ), LPS + LIQ40 group (LPS+40 mg/kg/d LIQ), LPS + LIQ80 group (LPS+80 mg/kg/d LIQ). Before LPS treatment (10 mg/kg, once, intraperitoneal injection), mice were administrated by gavage LIQ (dissolved in 60% ethanol and then diluted with 0.9% saline to the final concentration) or equal volumes of vehicle for 7 consecutive days. To examine the survival rate, mice were assigned into 4 separate study groups: CON, LIQ80, LPS (30 mg/kg) and LPS (30 mg/kg) +LIQ80.

AMPKα2 knockout mice (AMPKα2^−/−^) described previously (Zhang et al., 2018b) were chosen to study the underlying mechanism. Wild-type mice were also used for direct comparison. All mice were pre-treated with LIQ (80 mg/kg) for 7 days and were then injected with 10 mg/kg LPS to induce SCM.

### Echocardiography Analysis and Hemodynamics

Twelve hours following LPS administration, echocardiograph and hemodynamic studies were conducted to assess cardiac function as described previously (Zhang et al., 2018a). Mice were anesthetized by inhalation of isoflurane prior to echocardiography [Mylab 30CV (Esaote S.P.A, Genoa, Italy) with a 10 MHz linear array ultrasound transducer]. We selected and measured the short-axis standard view of the left ventricular papillary muscle. Heart rate (HR), left ventricular ejection fraction (LVEF), left ventricular fractional shortening (LVFS), left ventricular end-diastolic dimension (LVEDd), left ventricular end-systolic dimension (LVESd), end-diastolic interventricular septal thickness (IVSd), left ventricular posterior wall thickness (LVPWS) were recorded to evaluate mice cardiac function.

A microtip catheter transducer (SPR-839, Millar Instruments, Houston, TX, United States) was used to measure hemodynamic parameters. Mice were anesthetized with 1.5% isoflurane and the transducer was inserted into the left ventricle through the right carotid artery. Signals were continuously recorded using a Millar Pressure-Volume System (MPVS-400, Millar Instruments).

### Neonatal Rat Cardiomyocytes Isolation and Treatment

Neonatal rat cardiomyocytes (NRCM) were prepared as described previously (Zhang et al., 2018a). Briefly, 1–3 days old neonatal SD rats were sacrificed by dislocation, the heart removed and cut into small pieces. Tissue was digested with 0.125% trypsin, centrifuged, the supernatant collected and filtered, and then the filtered cell suspension was seeded into a plate. After 2 hours, the myocyte count in the supernatant was measured using a hemocytometer, and then myocytes were seeded into 6, 24 or 96 well plates, and incubated in a 5% CO_2_ incubator at 37°C with Dulbecco’s modified Eagle medium (DMEM) (GIBCO, C11995) supplemented with 15% fetal bovine serum (FBS) and 0.1 mM (E)-5-(2-bromovinyl)-2′-deoxyuridine (Brdu) to inhibit fibroblast growth. After pre-treating with 40 μM LIQ or an equal volume of PBS for 2 hours, 1 μg/ml LPS was added to cells for incubating 12 hours.

To further confirm that LIQ protected against LPS-induced cardiomyocyte injuries via an AMPKα dependent mechanism, Compound C (CC) was used to inhibit AMPKα in NRCM. NRCMs were pre-incubated with CC (10 μM) and LIQ (40 μM) for 2 hours before LPS treatment. Cells were assigned into five groups, CON, LIQ group, LPS group, LPS + LIQ group and LPS + LIQ + CC group. CCK8 was used to examine cell viability according to the manufacturer’s protocol.

### Western Blotting

Heart tissues and NRCMs were homogenized with RIPA lysis buffer and quantified by BCA assay kit (Thermo, Waltham, MA, United States). 50 μg protein was loaded into 8%, 10% or 12% SDS-PAGE gels according to different molecular weights and transferred to polyvinylidene difluoride (PVDF) membranes (Millipore, Billerica, MA, United States). After blocking with 5% non-fat milk, blots were incubated with the primary antibodies overnight at 4°C. The next day, blots were incubated with peroxidase-labeled secondary antibody (LI-COR Biosciences, 1:10,000 dilution) at room temperature for 1 hour, and signals were detected with a chemiluminescence ECL kit (Bio-Rad, United States). The results of each blot were analyzed by Image Lab 5.2.1. All proteins were normalized to GAPDH before relative quantitative analysis.

### Real-Time Reverse Transcriptase-Polymerase Chain Reaction Analysis

Heart tissue or NRCMs were lyzed in Trizol (Invitrogen, Carlsbad, CA, United States) for RNA extraction. 2 μg of total RNA was reverse transcribed into cDNA by oligo (dT) primers using a cDNA synthesis kit (Roche, Mannheim, Germany). The expression level of mRNA was quantified with SYBR green, and mRNA expression of GAPDH was used as an internal reference before relative quantitative analysis. Primers used in this study were shown in Table 1.

**TABLE 1:** Primers used for RT-PCR.

Gene	Species	Forward primer (5′→3′)	Reverse primer (5′→3′)
IL-1β	Mouse	CCG​TGG​ACC​TTC​CAG​GAT​GA	GGG​AAC​GTC​ACA​CAC​CAG​CA
IL-6	Mouse	AGT​TGC​CTT​CTT​GGG​ACT​GA	TCC​ACG​ATT​TCC​CAG​AGA​AC
TNFα	Mouse	CAT​CTT​CTC​AAA​ATT​CGA​GTG​ACA​A	TGG​GAG​TAG​ACA​AGG​TAC​AAC​CC
PGC1α	Mouse	CCG​AGA​ATT​CAT​GGA​GCA​AT	GTG​TGA​GGA​GGG​TCA​TCG​TT
PGC1β	Mouse	AGC​GCT​TTG​AGG​TGT​TCG​GTG​A	AGG​AGG​GCT​CAT​TGC​GCT​TTC​T
GAPDH	Mouse	ACT​CCA​CTC​ACG​GCA​AAT​TC	TCT​CCA​TGG​TGG​TGA​AGA​CA
IL-1β	Rat	GGG​ATG​ATG​ACG​ACC​TGC​TAG	ACC​ACT​TGT​TGG​CTT​ATG​TTC​TG
IL-6	Rat	GAT​GTT​GTT​GAC​AGC​CAC​TGC	GTC​TGT​TGT​GGG​TGG​TAT​CCT
TNFα	Rat	AGC​ATG​ATC​CGA​GAT​GTG​GAA	TAG​ACA​GAA​GAG​CGT​GGT​GGC
GAPDH	Rat	TCT​CTG​CTC​CTC​CCT​GTT​CTA	CTA​AAC​CCG​TAC​AGC​GTC​CT

### Terminal Deoxynucleotidyl Transferase-Mediated dUTP Nick-End-Labeling Staining

To analyze myocardial apoptosis, Terminal deoxynucleotidyl transferase-mediated dUTP nick-end-labeling (TUNEL) staining was performed with a commercial kit (Millipore, Billerica, MA, United States) according to the manufacturer’s instructions, and then the images were assessed by Image Pro Plus 6.0 (Maryland, United States).

### Oxidative Stress Examination

Total superoxide dismutase (SOD) Assay Kit with WST-8 (S0101) was purchased from Beyotime Co. (Shanghai, China) and the assay was performed according to the manufacturer’s instructions. The activity of NADPH oxidase was detected by commercial kits (Shanghai, China).

### Enzyme-Linked Immunosorbent Assay

Mouse IL-6 ELISA kits (Biolegend, 341303), Mouse TNF-α ELISA kits (Biolegend, 430906), and Mouse IL-1β ELISA kits (Biolegend, 432603) were used to examine the level of inflammatory cytokines in mouse heart. The assays were done according to the manufacturer's instructions.

### Immunohistochemistry Staining

Hearts were harvested and arrested in 10% KCL, then embedded in paraffin and cut into 10 μm sections. After antigen repairing in a pressure cooker, the heart tissue sections were incubated with 3% H_2_O_2_ for 10 minutes, and then TBST was used for tissue blocking for 1 hour prior to incubation with primary antibodies against CD45 (#ab10558) and CD68 (#ab125212). Tissues were incubated overnight at 4°C before application of the secondary antibody, followed by the use of DAB to visualize staining.

### Statistical Analysis

All data are presented as Means ± SEM for the number (*n*) of experiments. A log-rank test was used to compare survival curves. Differences among three or more groups were analyzed by one-way ANOVA with Tukey post hoc test. Data was statistically significant when *p* < 0.05. All experiments were done randomized and blinded if it is necessary.

## Results

### Liquiritin Attenuates Lipopolysaccharides-Induced Mouse Cardiac Dysfunction

**FIGURE 1:**
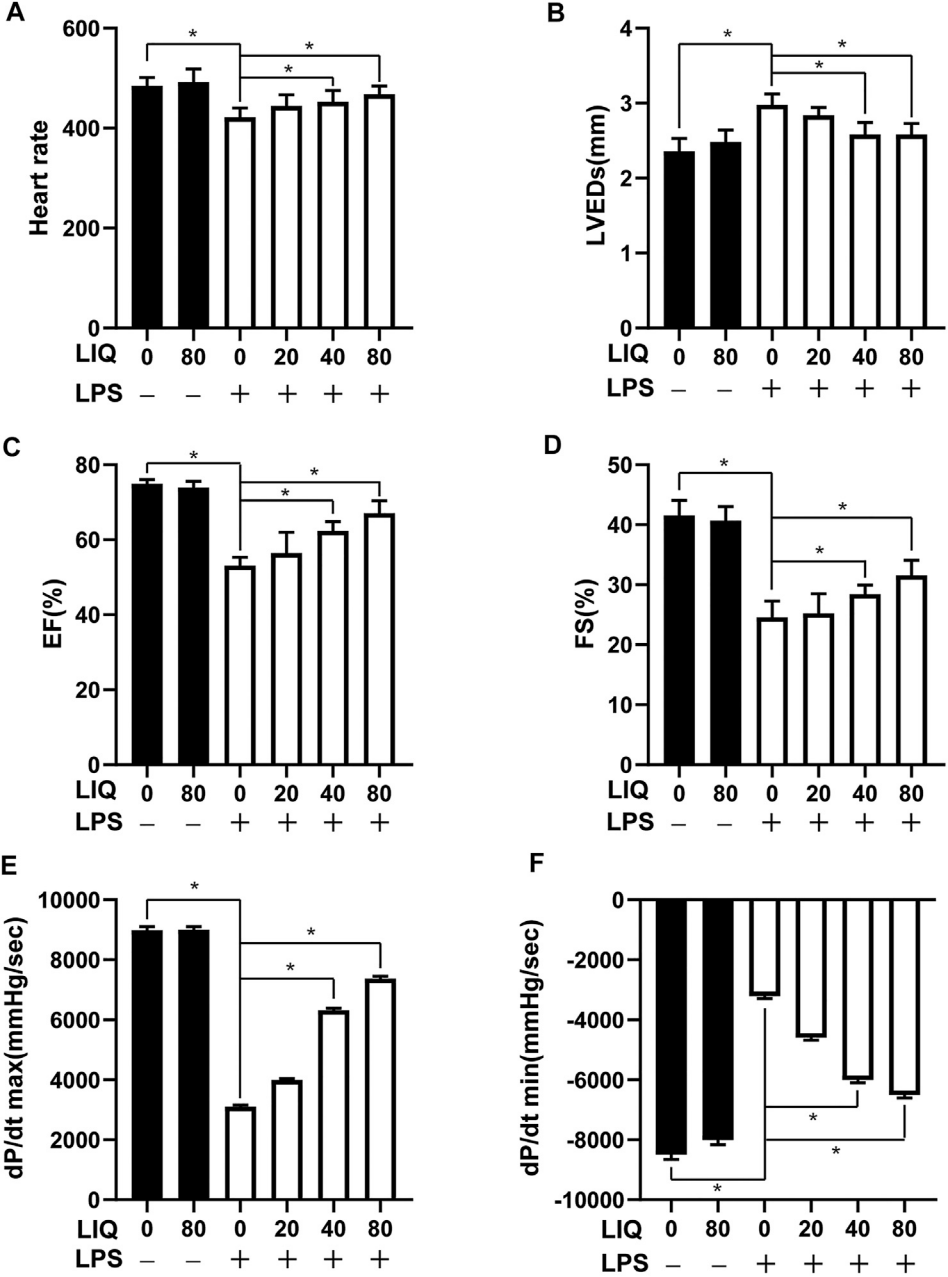
LIQ treatment attenuated LPS-induced mouse cardiac dysfunction Echocardiography and pressure-volume loop was performed to analyze mouse cardiac function after treatment with LPS (10 mg/kg) or saline for 12 hours, **(A)** heart rate (HR), **(B)** left ventricular end-systolic diameter (LVEDs), **(C)** left ventricular ejection fraction (EF), **(D)** left ventricular fractional shortening (FS), **(E)** maximal rate of pressure development (dp/dt max), **(F)** minimal rate of pressure decay (dp/dt min), (*n* = 10). The data were expressed as the Mean ± SEM. ∗*p* < 0.05 compared with the indicated group. The data were compared by one-way ANOVA with Tukey post hoc analysis.

Mice injected with LPS (10 mg/kg) presented with symptoms of cardiac dysfunction evidenced by increased LVEDs and decreased HR, EF and FS in LPS group compared with the CON or LIQ groups (Figures 1A–D). LIQ administration (40 mg/kg/d and 80 mg/kg/d) attenuated LPS-induced cardiac dysfunction. However, the lower LIQ dose (20 mg/kg/d) did not significantly protect against LPS-induced cardiac dysfunction compared with the CON or LIQ groups (Figures 1A–D). Pressure-volume loop analysis was performed to further examine mouse cardiac function. LPS injection significantly reduced dp/dt max and dp/dt min compared to CON or LIQ group (Figures 1E,F). LIQ treatment (40 mg/kg/d and 80 mg/kg/d) markedly restored dp/dt max and dp/dt min compared to LPS group (Figures 1E,F). The lower LIQ dose (20 mg/kg/d) showed no significant effect for dp/dt max and dp/dt min compared to LPS group Taken together, these data suggested that LIQ administration ameliorated LPS-induced mouse cardiac dysfunction.

### Liquiritin Suppressed Lipopolysaccharides-Induced Cardiac Inflammation *In Vivo*

**FIGURE 2:**
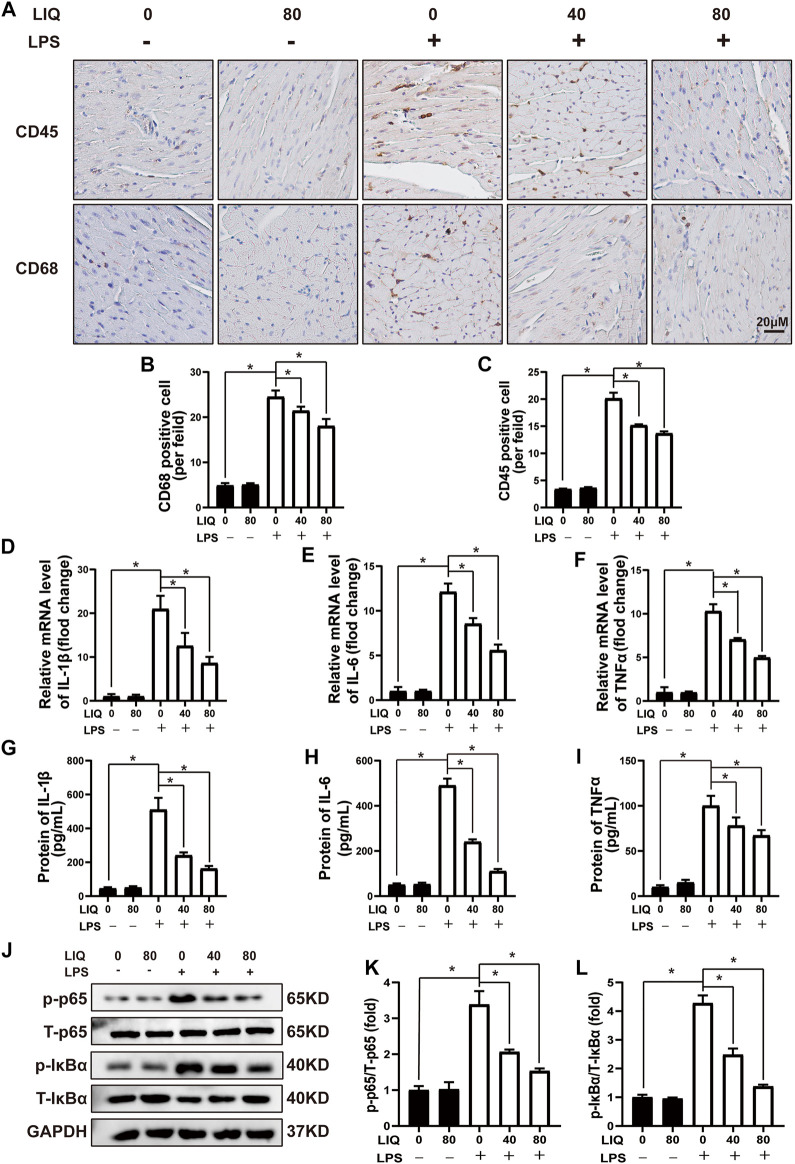
LIQ treatment suppressed LPS-induced cardiac inflammation *in vivo* **(A)** Immunohistochemical staining was performed to detect CD-45 and CD-68 in mouse left ventricle (*n* = 6), **(B,C)** Counting CD68 and CD45 positive cells (*n* = 6), **(D–F)** RT-PCR was performed to examine mRNA expression of IL-1β, IL-6 and TNFα (*n* = 6), **(G–I)** ELISA was performed to detect proteins expression of including IL-1β, IL-6 and TNFα (*n* = 6), **(J–L)** Representative western blots and relative quantitative results of proteins in NF-κB signaling pathway, including phosphorylated P65 (p-P65), total P65 (T-P65), p-IκBα and T-IκBα (*n* = 6). Mice were treated with a dose of 10 mg/kg LPS, the data are expressed as the Mean ± SEM. ∗*p* < 0.05 compared with the indicated group. The data were compared by one-way ANOVA with Tukey post hoc analysis.

LPS treatment caused accumulation of CD45-labeled leukocytes and CD68 positive cells in heart tissue (Figures 2A–C), which was effectively attenuated by 40 mg/kg/d or 80 mg/kg/d of LIQ treatment (Figures 2A–C). Moreover, IL-1β, IL-6 and TNFα expression was significantly up-regulated in LPS-treated mice hearts compared to the CON or LIQ group (Figures 2D–I), and LIQ treatment significantly depressed the expression of IL-1β, IL-6 and TNFα compared to LPS injection group (Figures 2D–I).

We also measured IκBα/NF-κB signaling pathways that play important roles in regulating inflammatory responses. The data demonstrated that phosphorylated p65/IκBα was significantly up-regulated, and T-IκBα was significantly down-regulated by LPS treatment (Figures 2J–L). LIQ (40 mg/kg/d or 80 mg/kg/d) treatment significantly inhibited the p65 and IκBα phosphorylation and restored the T-IκBα expression compared to LPS group (Figures 2J–L).

### Liquiritin Treatment Suppresses Lipopolysaccharides-Induced Oxidative Stress and Apoptosis *In Vivo*

LPS treatment significantly increased NADPH oxidase activity and decreased SOD activity in LPS-treated mice compared to the CON or LIQ groups (Supplementary Figure S1). LIQ treatment (40 mg/kg/d or 80 mg/kg/d) restored SOD activity to normal and decreased NADPH oxidase activity in LPS + LIQ mice (Supplementary Figure S1). The results show that LIQ treatment ameliorates LPS-induced oxidative stress.

**FIGURE 3:**
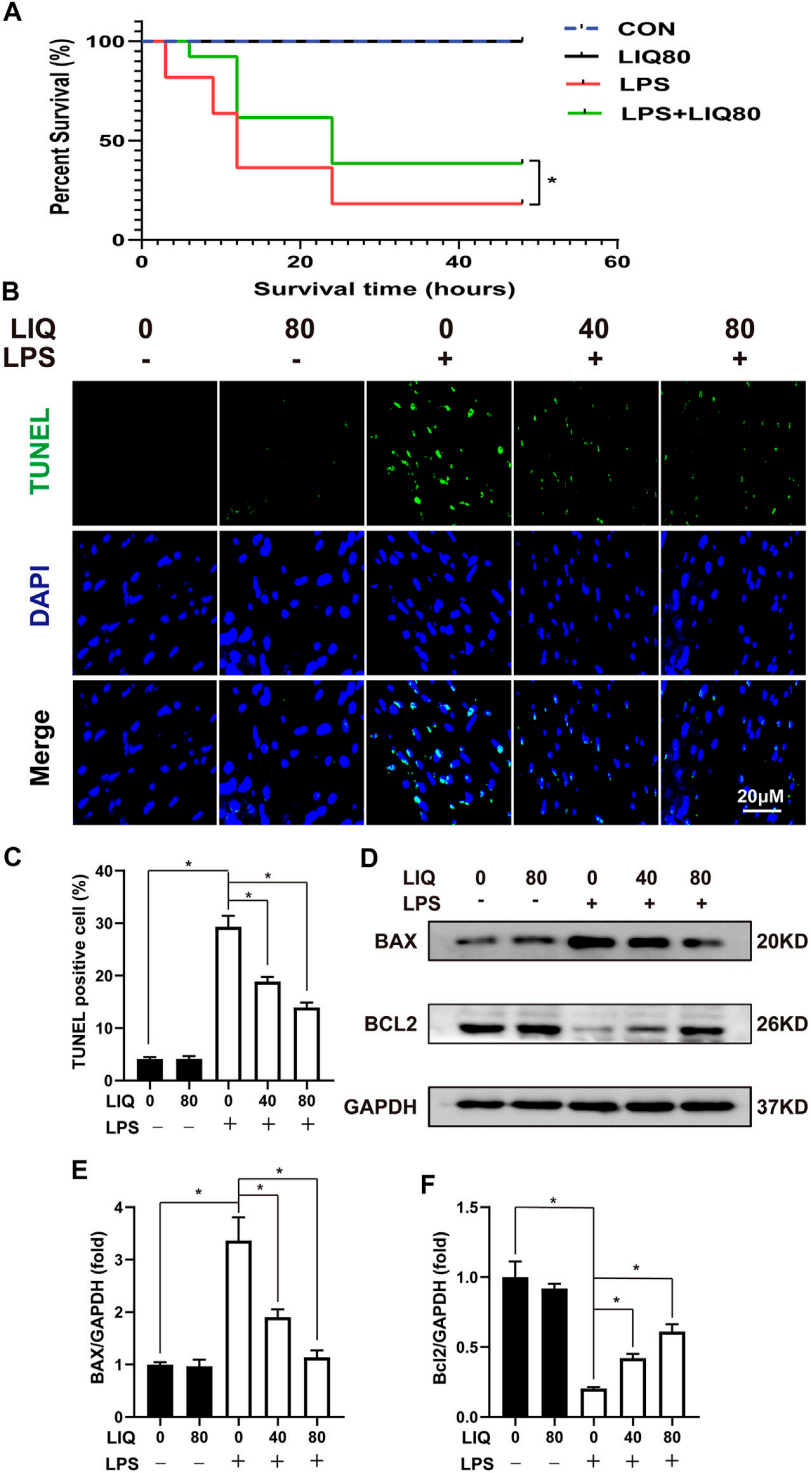
LIQ treatment suppressed LPS-induced oxidative stress and apoptosis *in vivo*. **(A)** Mice were treated with LPS (30 mg/kg, intraperitoneal injection) for observing 48 h to draw survival curves (*n* = 20), **(B)** TUNEL staining was performed to examine cellular apoptosis in mouse hearts (*n* = 6), **(C)** Calculating the TUNEL positive nucleus (*n* = 6), **(D)** Representative western blots of BAX, Bcl-2 and GAPDH, **(E,F)** relative quantitative results of BAX/GAPDH and Bcl-2/GAPDH (*n* = 6). *p < 0.05 compared with the indicated group, the data were expressed as the Mean ± SEM and were compared by one-way ANOVA with Tukey post hoc analysis, A log-rank test was used to compare survival curves.

LPS (30 mg/kg) treatment significantly increased mouse mortality rate compared to the CON or LIQ groups (Figure 3A). LIQ (80 mg/kg) treatment significantly prevented LPS-induced mortality (Figure 3A). Cardiomyocyte loss appears to play an important role in cardiac dysfunction and increased mortality following LPS administration. As shown in Figures 3B,C, TUNEL staining indicated that LPS (30 mg/kg) treatment significantly induced cell apoptosis compared to CON. BAX, the pro-apoptosis protein, was significantly increased, but BCL2, the anti-apoptosis protein, was significantly decreased by LPS treatment in LPS group compared to CON or LIQ group (Figures 3D–F). LIQ (40 mg/kg/d or 80 mg/kg/d) treatment significantly reduced TUNEL positive cardiomyocytes, decreased BAX expression and restored Bcl2 expression (Figures 3B–F). These results indicated that LIQ treatment effectively reduced LPS-induced cardiomyocyte apoptosis.

### Liquiritin Treatment Regulates AMPKα2/mTOR Signaling

**FIGURE 4:**
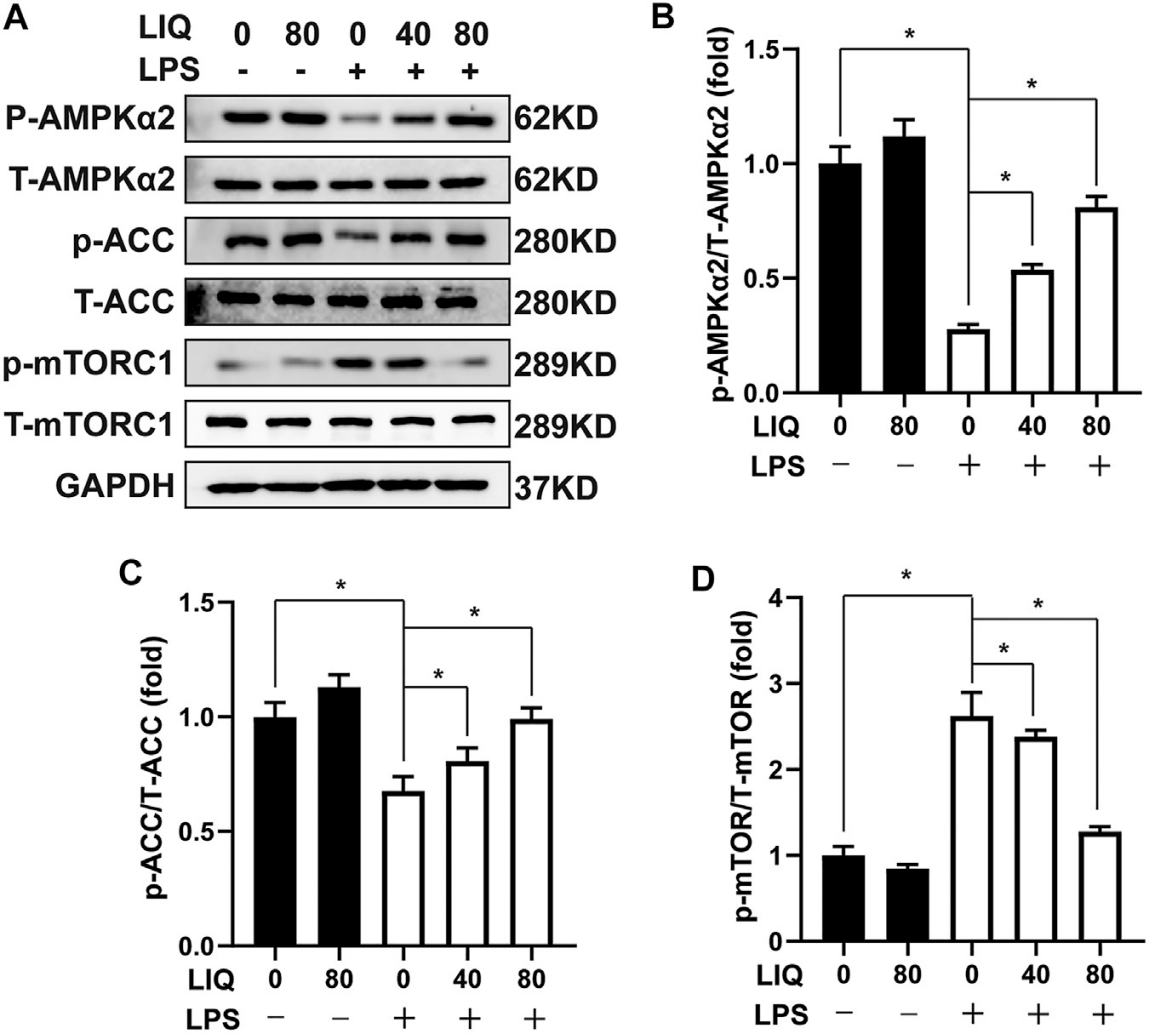
LIQ treatment regulated AMPKα2/mTOR signaling **(A)** Representative western blots of phosphorylated AMPKα2 (p-AMPKα2), total AMPKα2 (T-AMPKα2), p-ACC, T-ACC, p-mTORC1, T-mTORC1 and GAPDH, mouse hearts were collected for western-blots analysis after treated with or without LPS (10 mg/kg) for 12 h (*n* = 6), relative quantitative of **(B)** (p-AMPKα2/T-AMPKα2), **(C)** p-ACC/T-ACC and **(D)** p-mTORC1/T-mTORC1 (*n* = 6), all of these proteins were normalized to GAPDH before the relative quantitative analysis. *p < 0.05 compared with the indicated group, the data are expressed as the Mean ± SEM and were compared by one-way ANOVA with Tukey post hoc analysis.

We examined several signaling pathways including MAPK, AKT/GSK3β, JAK/STAT and AMPKα2/mTOR pathway to elucidate the underlying mechanisms of LIQ treatment. LIQ treatment showed to regulate the AMPKα2/mTOR pathway, but none of the other pathways (Supplementary Figure S2). LPS treatment significantly decreased AMPKα2 phosphorylation accompanied by decreased ACC phosphorylation but increased mTORC1 phosphorylation compared to the CON or LIQ group (Figures 4A–D). LIQ treatment restored AMPKα2 and ACC phosphorylation and inhibited mTORC1 phosphorylation in LPS + LIQ group compared to the LPS group (Figures 4A–D).

### Liquiritin Treatment Restores Expression of Fatty Acid Metabolism Associated Proteins

**FIGURE 5:**
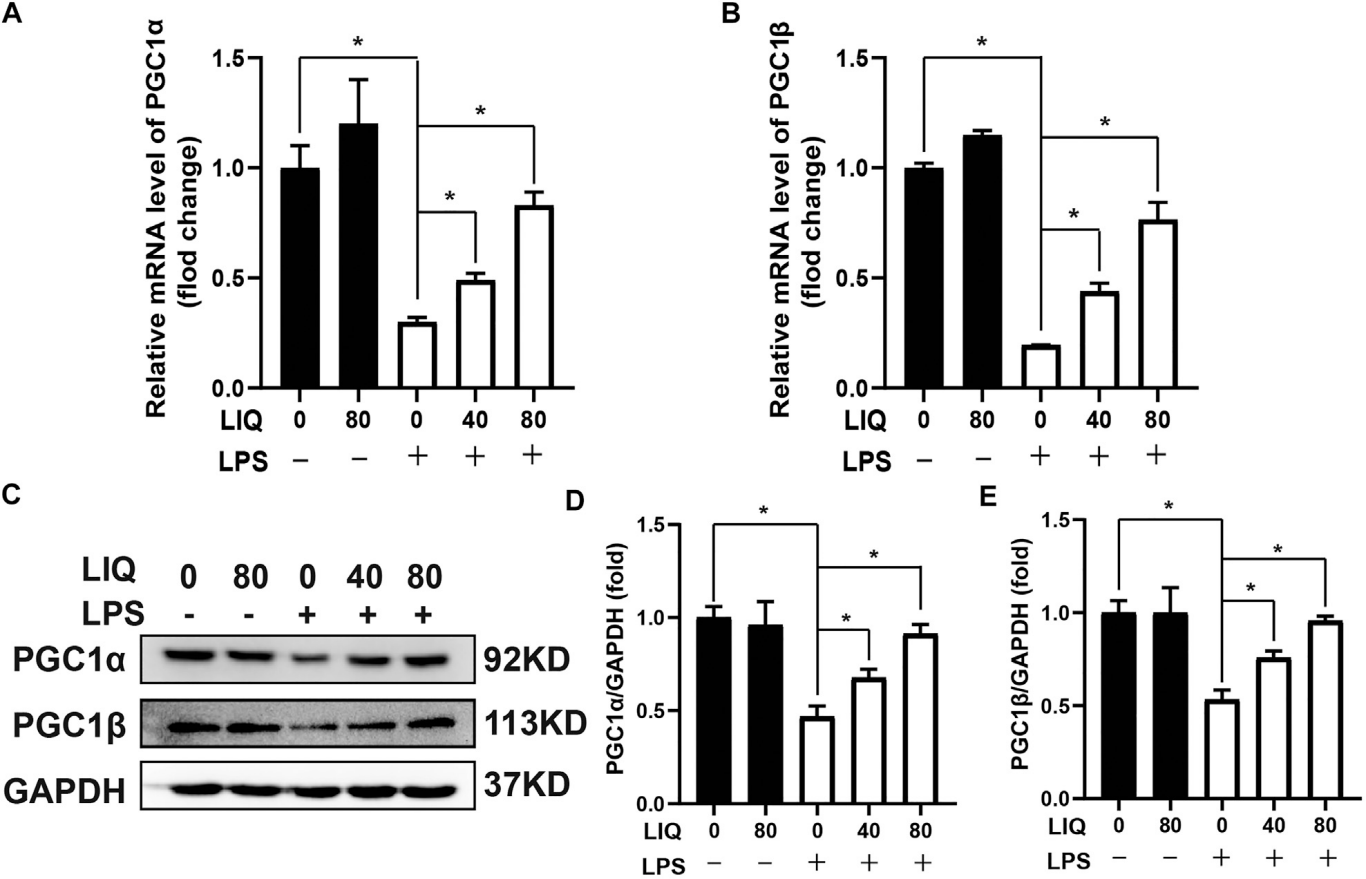
LIQ treatment improved fatty acid metabolism. RT-PCR was performed to analyze the mRNA expression of fatty acid metabolism associated genes in mouse hearts, including **(A)** PGC1α, **(B)** PGC1β, mRNA of GAPDH was used as internal reference (*n* = 6), **(C)** Representative western blots images of PGC1α, PGC1β and GAPDH, relative quantitative analysis of PGC1α/GAPDH **(D)**, PGC1β/GAPDH **(E)** (*n* = 6). Data were presented as the Mean ± SEM. *p < 0.05 compared with the indicated group, the data were expressed as the Mean ± SEM and were compared by one-way ANOVA with Tukey post hoc analysis.

AMPKα is a conserved sensor of cellular energy change. Phosphorylated AMPKα could maintain energy balance by decreasing anabolism processes but promoting catabolism to preserve ATP production when senses enhancement of AMP/ATP or ADP/ATP ratios (Ke et al., 2018). LPS treatment decreased the mRNA expression of PGC1α and PGC1β (Figures 5A,B), which are involved in regulating mitochondrial metabolism. LIQ treatment restored mRNA expression of PGC1α and PGC1β. (Figures 5A,B). LPS treatment also significantly down-regulated the protein expression of PGC1α and PGC1β compared to the CON or LIQ groups (Figures 5C–E). LIQ treatment prevented LPS-induced down-regulation of these proteins (Figures 5C–E).

### Liquiritin Treatment Showed No Protective Effects in Lipopolysaccharides Treated AMPKα2 Knockout Mice

**FIGURE 6:**
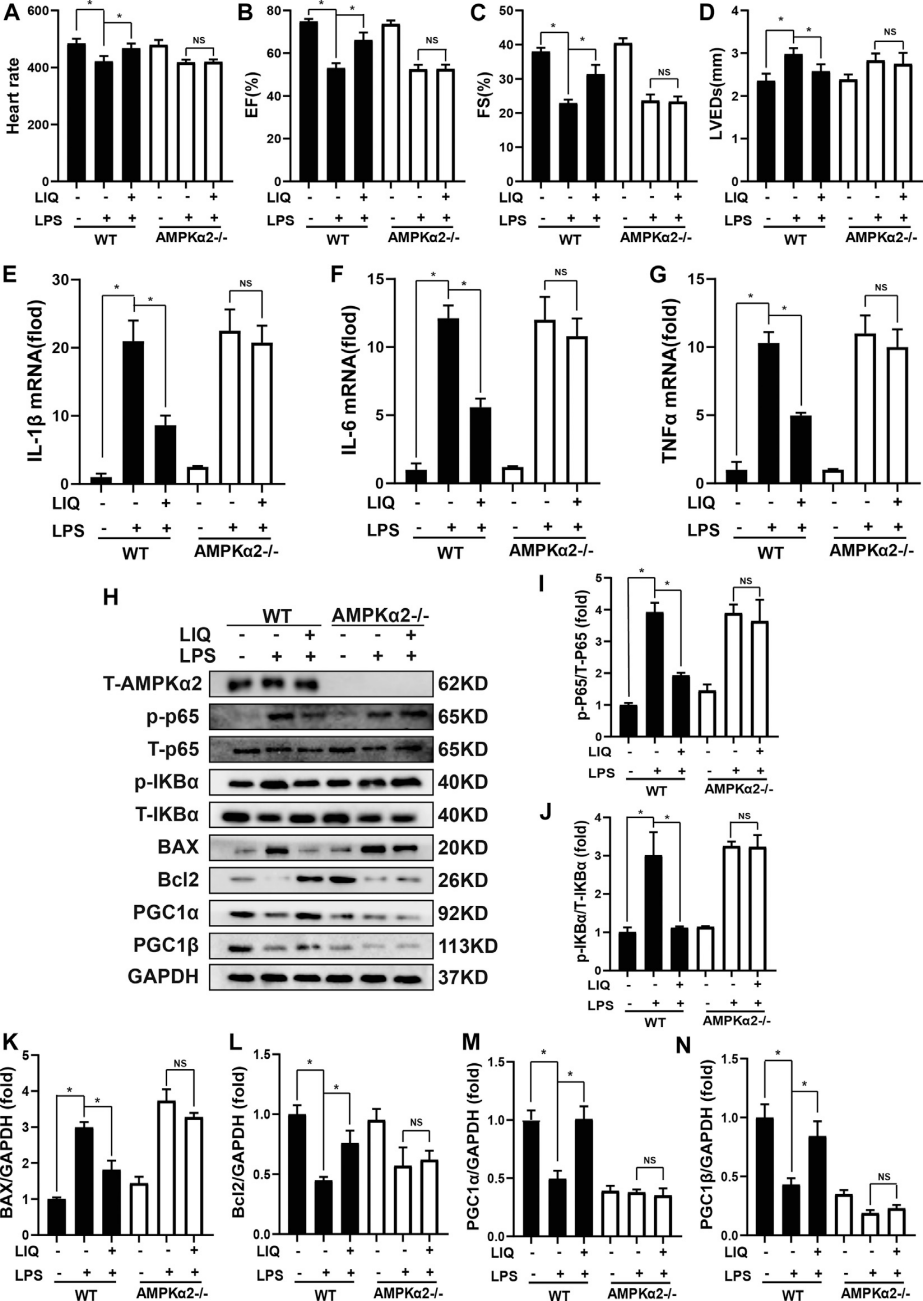
LIQ treatment could not prevent LPS-induced mouse heart injuries after AMPKα2 knockout Echocardiography was performed to analyze **(A)** Heart rate, **(B)** left ventricle ejection fraction (EF), **(C)** left ventricular fractional shortening (FS) **(D)** left ventricular end-systolic diameter (LVEDs) (*n* = 12). RT-PCR was performed to detect the mRNA expression of **(E)** IL-1β, **(F)** IL-6 and **(G)** TNFα (*n* = 6), all of mRNAs expression were normalized to GAPDH before the relative quantitative analysis, **(H)** Representative western-blots of T-AMPKα2, p-P65, T-P65, p-IκBα, T-IκBα, BAX, Bcl2, PGC1α, PGC1β and GAPDH (*n* = 6), relative quantitative analysis proteins expression of **(I)** p-P65/T-P65, **(J)** p-IκBα/T-IκBα, **(K)** BAX/GAPDH, **(L)** Bcl2/GAPDH, **(M)** PGC1α/GAPDH and **(N)** PGC1β/GAPDH, all proteins were normalized to GAPDH before the quantitative analysis (*n* = 6). AMPKα2 knockout mice were pretreated with LIQ (80 mg/kg/d) for 7 days and then were injected with LPS (10 mg/kg) for 12 h before these experiments. *p < 0.05 compared with the indicated group, the data were expressed as the Mean ± SEM and were compared by one-way ANOVA with Tukey post hoc analysis.

AMPKα2 knockout mice (AMPKα2−/−) were used to further determine whether LIQ treatment could prevent LPS-induced cardiac injury. AMPKα2−/− mice were pre-treated with LIQ (80 mg/kg/d) for 7 days and then were injected with LPS (10 mg/kg) for 12 hours. LIQ treatment neither effectively reversed EF, FS, LVEDs nor inhibit LPS-induced myocardial mRNA levels of IL-1β, IL-6, TNFα (Figures 6E–G), nor inhibit p65 and IKBα phosphorylation and Bax expression, and nor restored Bcl2, PGC1α, and PGC1β expression in AMPKα−/−mouse heart (Figures 6H–N). These results suggested that the protective roles of LIQ depend upon activating AMPKα activity.

### Liquiritin Treatment Didn’t Protect Against Lipopolysaccharides-Induced NRCMinjury After Inhibiting AMPKα Activity *In Vitro*

**FIGURE 7:**
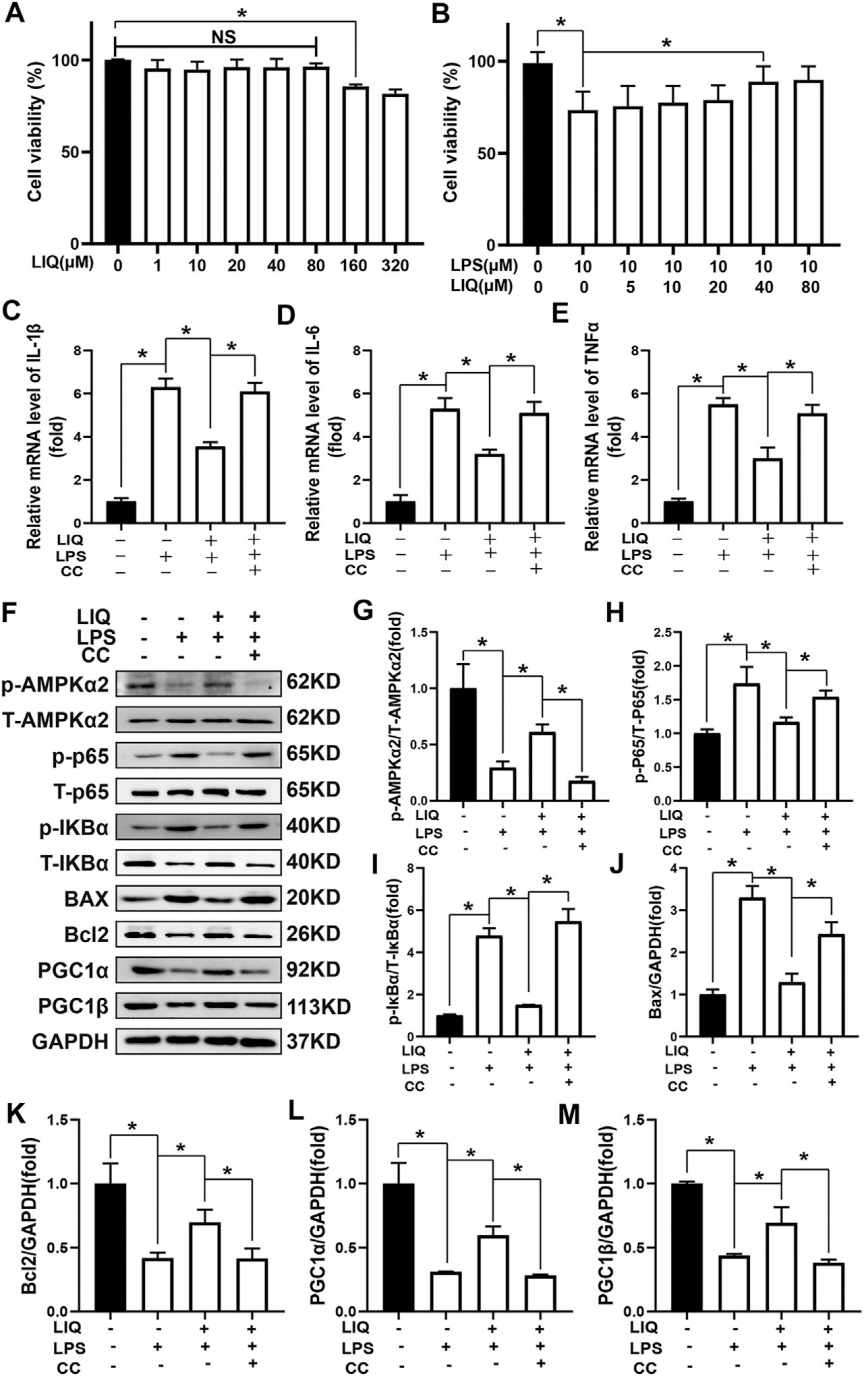
LIQ treatment could not protect against LPS-induced cardiomyocyte injuries after inhibiting AMPK activity *in vitro* **(A)** CCK-8 kit was used to examine cell viability after treating different concentration of LIQ (0, 1, 10, 20, 40, 80, 160, 320 µM) for 12 h (*n* = 9), **(B)** CCK-8 was used to examine cell viability in different treatment as indicated in the picture (*n* = 9), NRCMs were pretreated with LIQ for 2 h and then were treated with LPS (1 μg/ml) for another 12 h, RT-PCR was performed to analyze mRNA expression of IL-1β **(C)**, IL-6 **(D)** and TNF-α **(E)**, all of the mRNA expression were normalized to GAPDH before the relative quantitative analysis (*n* = 6). **(F)** representative western-blots of phosphorylated AMPKα2 (p-AMPKα2), total-AMPKα2 (T-AMPKα2), p-P65, T-P65, p-IκBα, T-IκBα, BAX, Bcl2, PGC1α, PGC1β and GAPDH (*n* = 6), relative quantitative analysis proteins expression of p-AMPKα2/T-AMPKα2 **(G)**, p-P65/T-P65 **(H)**, p-IκBα/T-IκBα **(I)**, BAX/GAPDH **(J)**, Bcl2/GAPDH **(K)**, PGC1α/GAPDH **(L)** and PGC1β/GAPDH **(M)** (*n* = 6), all proteins were normalized to GAPDH before the quantitative analysis. The cell experiment was repeated 3 times independently. *p < 0.05 compared with the indicated group, the data were expressed as the Mean ± SEM and were compared by one-way ANOVA with Tukey post hoc analysis.

NRCMs were pre-treated with LIQ (0, 1, 10, 20, 40, 80, 160, 320 μM) for 24 h. LIQ (≤80 μM) exhibited no cytotoxicity (Figure 7A). In addition, we pre-treated with LIQ (0, 5, 10, 20, 40, 80 μM) for 3 h and then treated with or without 1 μg/ml LPS for another 12 h. LIQ pretreatment with 40 or 80 μM of LIQ significantly preserved cardiomyocyte viability compared with LPS treatment group (Figure 7B). LPS (1 μg/ml) treatment significantly increased mRNA expression of IL-1β, IL-6 andTNFα in NRCMs, which could be effectively depressed by LIQ treatment, but LIQ could not depressed mRNA expression of these inflammatory cytokines after CC treatment (Figures 7C–E). LPS treatment inhibited AMPKα2 phosphorylation but promoted IκBα and p65 phosphorylation and down-regulated Bcl2, PGC1α and PGC1β expression but enhanced BAX expression (Figures 7F–M). LIQ treatment restored AMPKα2 phosphorylation, inhibited IκBα, p65 phosphorylation and BAX expression and restored Bcl2, PGC1α and PGC1β expression (Figures 7F–M). None of these beneficial effects of LIQ were observed in the presence of CC (Figures 7F–M).

## Discussion

This study demonstrated that LIQ treatment could prevent LPS-induced cardiomyocyte injury *in vivo* and *in vitro* by regulating AMPKα2 and IκBα/NF-κB signaling pathway. This study suggested that LIQ may be a useful therapy or adjuvant therapy for SCM. SCM may occur in sepsis patients with a morbidity rate as high as 60% (Rudiger and Singer, 2013). SCM is characterized by left ventricular dilation with low filling pressure and significantly reduced ejection fraction (Beesley et al., 2018). Even though SCM could be reversible within 7–10 days from onset, acute myocardial depression in SCM might cost patient’s life.

It has been demonstrated that cytokines, especially IL-1β, IL-6, and TNF-α, are major contributors to the initiation of SCM (Drosatos et al., 2015). In an endotoxin model, cardiac tissue is infiltrated by immune cells, such as lymphocytes, monocytes and macrophages. These immune cells could secret inflammatory cytokines to further exaggerate inflammatory response. LPS is a major component of the outer membrane of Gram-negative bacteria and is used to establish sepsis shock and to mimic SCM-associated heart injury in mice (Chen et al., 2017). Our result showed that LPS treatment significantly induced IL-1β, IL-6 and TNF-α accumulation and immune cell infiltration, which could be effectively inhibited by LIQ treatment. This was consistent with previous studies that showed that LIQ exerts its anti-inflammatory effect in several disease models via inhibiting the NF-κB and NLRP3 inflammasome pathways (Min et al., 2015; Yu et al., 2015; Hongyan et al., 2016). We confirmed here that LIQ treatment could inhibit IκB/NF-κB signaling, which was in agreement with previous studies.

Oxidative stress is another very important mechanism involved in SCM pathogenesis, and increased oxidative stress correlates with SCM severity and mortality (Tsolaki et al., 2017). Endotoxin treatment may inhibit antioxidant enzyme activity and promote oxidative stress-associated injury and apoptosis. Matsuno showed that the NADPH subunit (NOX1) was up-regulated in SCM, which was accompanied by excessive ROS production and cardiomyocyte apoptosis (Matsuno et al., 2012). NOX1 deficiency significantly reduced LPS-induced cardiomyocyte apoptosis and improved mouse cardiac function (Matsuno et al., 2012). After the bacterial challenge, Zang also demonstrated sepsis-induced progressive oxidative damage and cytochrome C release in mitochondria accompanying with downregulation of SOD and GPx activity (Zang et al., 2007). Thus, the up-regulation of NADPH activity and down-regulation of SOD activity could exaggerate cardiomyocyte apoptosis and cardiac dysfunction. Our study showed that LIQ treatment could decrease NADPH activity but increase SOD activity, which could prevent cardiomyocytes from LPS-induced injuries and apoptosis.

SCM is also accompanied by decreased fatty acid oxidation (FAO), which accounts for more than 70% of the energy supply in adult cardiomyocytes (Beesley et al., 2018). A profile of key proteins regulating FAO has been down-regulated in the heart following LPS treatment. LPS or danger-associated molecular patterns (DAMPs) mediated toll-like receptor 4 (TLR4) activation causes PGC-1α and PGC-1β downregulation and mitochondrial energy metabolism disorder and the production of ROS in the heart (Schilling et al., 2011; Martin et al., 2015). Restoration of PGC1α and PGC1β expression could protect against LPS-TLR4 activation and metabolic alterations (Schilling et al., 2011; Martin et al., 2015). PGC1α has also been described as an important regulator of mitochondrial biogenesis (Ventura-Clapier et al., 2008); indicating that fine-tuning PGC1α expression might be an effective strategy to restore mitochondrial homeostasis, which could protect against pathological cardiac impairment (Zhu et al., 2019). Thus, promoting the expression of these down-regulated proteins might protect against LPS-induced cardiomyocyte energy depletion. Our study demonstrated that LIQ treatment could effectively reverse the down-regulation of proteins involved in cardiac metabolism.

Jung et al. reported that LIQ treatment could protect hepatocytes from serum deprivation-induced oxidative stress-associated injury and mitochondrial dysfunction in mouse liver by activating AMPK (Jung et al., 2017). AMPK exerts a distinct and critical role in regulating malonyl-CoA levels and FAO in the heart. Malonyl-CoA locates at the outer mitochondria membrane where it could inhibit the uptake of fatty acids into the mitochondria. AMPK could phosphorylate acetyl-CoA carboxylase (ACC) and could also phosphorylate and activate malonyl-CoA decarboxylase (MCD) (Hopkins et al., 2003). ACC inhibition and MCD activation could reduce the synthesis of malonyl-CoA resulted in increased fatty acid uptake (Hopkins et al., 2003). In addition, AMPK may also regulate contraction-inducible FAT/CD36 translocation in cardiomyocytes to enhance long-chain fatty acid uptake (Luiken et al., 2003). Activated AMPK may promote mitochondrial biogenesis to adapt to chronic energy deprivation by increasing PGC-1α expression (Zong et al., 2002). Activated AMPK has also been shown to take part in regulating inflammation and oxidative stress in cardiomyocytes. AMPK activation could alleviate LDH activity, ROS content and Nox1 expression resulting in reduced apoptosis and improved cell viability in CoCl2 induced hypoxic injury in cardiomyocytes (Tong et al., 2012). Wang et al. (Wang et al., 2018) showed that AMPK activation prevented cardiomyocyte necroptosis by reducing ROS production, however, these protective effects were disappeared after CC treatment or AMPK dominant-negative (Wang et al., 2018). In the LPS-induced SCM model, AMPK activation could inhibit IKK*β*/IκBα/NFκB signaling and reduce the mRNA expression of TNF-α, IL-6 and monocyte chemoattractant protein-1 (MCP1) (Huang et al., 2018). Jun Ren et al.(Ren et al., 2016) demonstrated that adiponectin deficiency exacerbated LPS-induced cardiac dysfunction, intracellular Ca^2+^ defect, inflammation and apoptosis, because adiponectin deficiency depressed activation of CAMKKβ-AMPK-ACC resulted in mTORC1 hyperphosphorylation and AMPK mediated autophagy inhibition. However, administration of the AMPK activator AICAR or mTORC1 inhibitor rapamycin obliterated accentuated cardiac dysfunction, apoptosis and inflammatory response in adiponectin deficiency mouse. This study demonstrated that AMPK activation could attenuate LPS-induced mouse heart injuries at least partly from inducing AMPK mediated autophagy. Although we didn’t examine AMPK mediated autophagy induction, it would be a reasonable deduction that LIQ treatment attenuated LPS-induced mouse hear injuries partly depending on AMPK activation mediated autophagy induction. To sum up, these studies indicated that activated AMPK might involve in regulating multiple biological processes, including oxidative stress, cellular apoptosis, inflammation and energy metabolism.-. Our study demonstrated that LIQ could promote AMPKα2 phosphorylation resulted in attenuating LPS-induced myocardial injury and cardiac dysfunction. However, LIQ treatment could not protect against LPS-induced myocardial injury and cardiac dysfunction in AMPKα2^−/−^ mice. Thus, our study firstly demonstrated that LIQ exerted its protective role at least partly rely on activating AMPKα2 signaling pathways (the mechanic summary is shown in the mechanic diagram).

## Conclusion

This study exhibited that LIQ treatment attenuated LPS-induced mouse cardiac dysfunction *in vivo* and *in vitro* via reducing inflammation, oxidative stress, and apoptosis and improving metabolism. Relying on AMPKα2 knockout mice, This study also demonstrated that LIQ exerted its protective roles depending on activating AMPKα2 phosphorylation. Moreover, LIQ presented to be ineffective in protecting against LPS-induced NRCMs injury *in vitro* after AMPKα activity inhibition. Although gene knockout mice are important to confirm the potential target of some drugs, it is not entirely clear that LIQ only exerts its protective role by regulating AMPKα2, since the protective effects of LIQ on other pathways may be masked by the deterioration caused by AMPKα2. Moreover, our investigation indicated that some other pathways, including MAPK, AKT/GSK3β, and JAK/STAT, seemed not to involve in LIQ’s protective roles in septic cardiomyopathy.

LIQ might be a potential adjuvant drug for treating SCM and inflammation-associated cardiomyocyte injuries. However, additional studies are required to further elucidate mechanisms in improving cardiac remodeling following LIQ treatment. Specially, this study exhibited that LIQ might involve in regulating lipid metabolism. Although we discussed that LIQ-regulated lipid metabolism might be partly associated with activating AMPKα2, it will be more interesting and necessary to provide additional lipidomic data to support and strengthen this finding in future studies.

## Data Availability

The raw data supporting the conclusion of this article will be made available by the authors, without undue reservation.
