# Supplementary material for: Liquiritin Attenuates Lipopolysaccharides-Induced Cardiomyocyte Injury via an AMP-Activated Protein Kinase-Dependent Signaling Pathway
Source: Front Pharmacol. 2021 May 14;12:648688. doi: 10.3389/fphar.2021.648688 (PMC8162655; doi:10.3389/fphar.2021.648688)
Supplement: Supplementary file 1 [file DataSheet1.PDF]

Supplemantary Figure 1

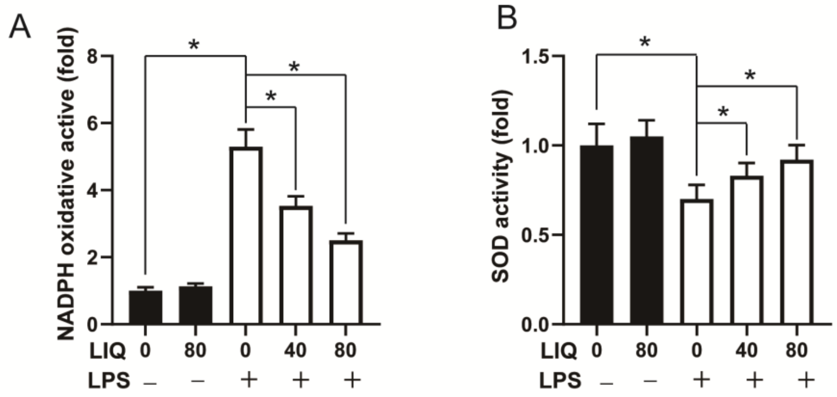

Supplemantary Figure 1: Oxidative stress associated commercial kits were used to examine (A) NADPH oxidase and (B) SOD activity in the heart tissue (n=6) *p < 0.05 compared with the indicated group, the data are expressed as the Mean ± SEM and were compared by one-way ANOVA with Tukey post hos analysis.

Supplemantary Figure 2

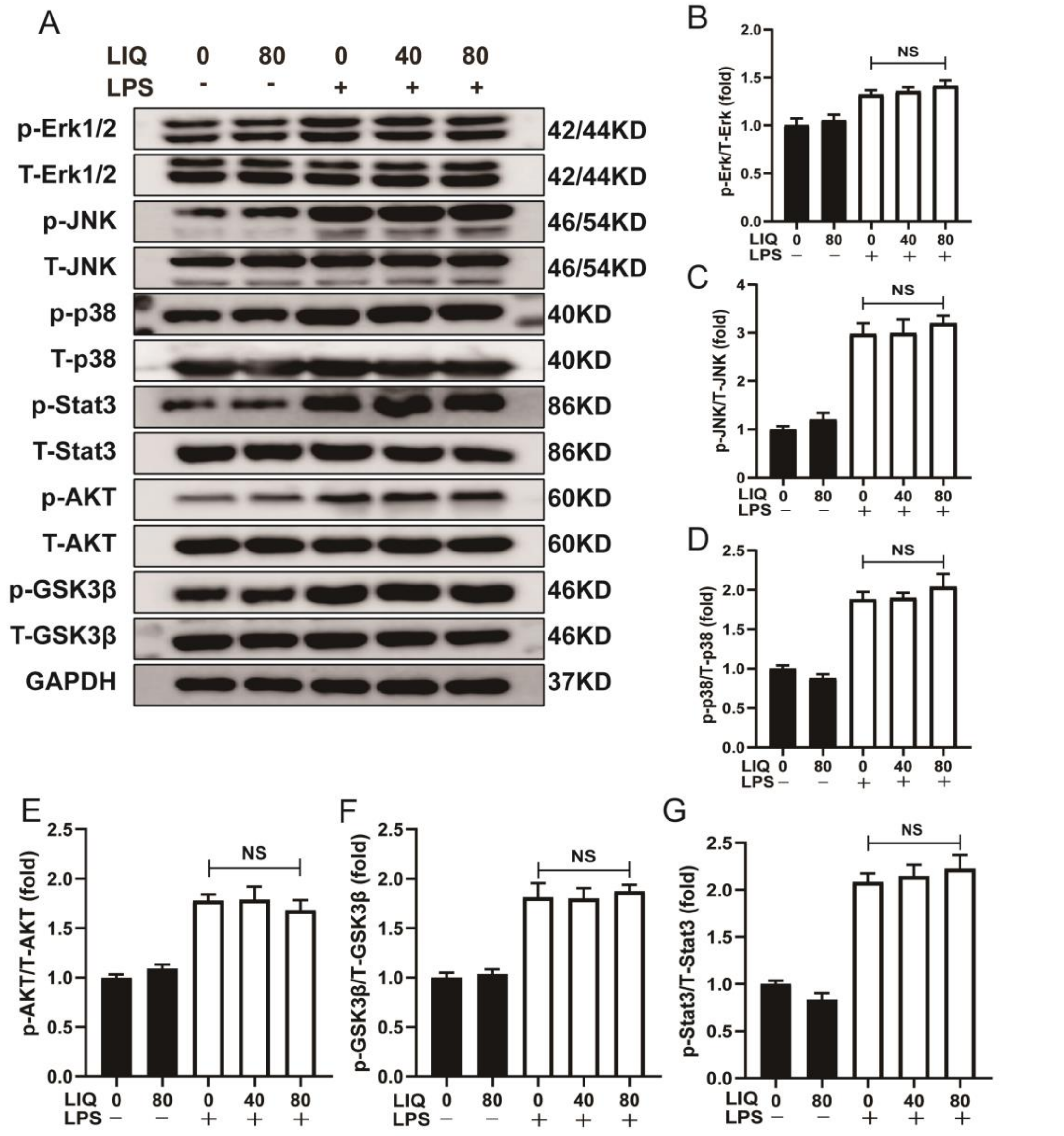

Supplemantary Figure 2： Pathways that did not change significantly under LIQ treatment

(A) Representative western blots of p-Erk, T-Erk, p-JNK, T-JNK, p-p38, T-p38, p-AKT, T-AKT, p-GSK3β, T-GSK3β, p-Stat3, T-Stat3, GAPDH, mouse hearts were collected for western-blots analysis after treated with or without LPS for 12 hours, relative quantitative of (B) p-Erk/T-Erk, (C) p-JNK/T-JNK, (D) p-p38/T-p38, (E) p-AKT/T-AKT, (F) p-GSK3β/T-GSK3β and (G) p-Stat3/T-Stat3, **p* < 0.05 compared with the indicated group, NS no significance, the data are expressed as the Mean ± SEM and were compared by one-way ANOVA with Tukey post hos analysis.
